# Targeted lipopolysaccharide biosynthetic intermediate analysis with normal-phase liquid chromatography mass spectrometry

**DOI:** 10.1371/journal.pone.0211803

**Published:** 2019-02-08

**Authors:** William S. Sawyer, Lisha Wang, Tsuyoshi Uehara, Pramila Tamrakar, Ramadevi Prathapam, Mina Mostafavi, Louis E. Metzger, Brian Feng, Christopher M. Baxter Rath

**Affiliations:** Infectious Diseases, Novartis Institutes for Biomedical Research, Emeryville, CA, United States of America; University of Missouri Columbia, UNITED STATES

## Abstract

Lipopolysacharride (LPS) forms the outer leaflet of the outer membrane in Gram-negative bacteria and contributes to the permeability barrier and immune response. In this study, we established a method for monitoring the LPS biosynthetic intermediates of the Raetz pathway (*lpxA-lpxK*) in *Escherichia coli*. Metabolites from compound-treated cells and genetically-perturbed cells were extracted from whole cells and concentrated by mixed-mode weak anion exchange (WAX) solid-phase extraction (SPE) prior to analysis by normal phase (NP)LC-MS/MS. Data was normalized to cell density and an internal standard prior to comparison against untreated cells in order to determine fold accumulation and depletion for affected metabolites. Using this LC-MS/MS method, we were able to reliably monitor changes in levels of the LPS intermediates in response to compound-treatment and genetic modification. In addition, we found that deletion of periplasmic CDP-diacylglycerol pyrophosphatase dramatically increased levels of the UDP-containing LPS intermediates, suggesting the enzymatic breakdown during sample preparation. This assay allows for probing a key essential pathway in Gram-negative bacteria in an effort to discover antibacterial agents that inhibit enzymes in the LPS biosynthetic pathway.

## Introduction

Due to the increasing rate of infections by multi-drug-resistant (MDR) Gram-negative bacterial pathogens, an urgent need for new antibiotics has become apparent[1]. Patients infected by these MDR bacterial strains, such as carbapenem-resistant *Enterobacteriaceae*, cannot be treated with available antibiotics[2]. Despite the dramatic reduction in effective treatment options for patients, there unfortunately has not been a substantial increase in discovery and development of novel antibiotics[3]. One of the biggest obstacles to overcome during target-based drug discovery is bridging the gap between *in vitro* biochemical and cellular activity. For example, enzyme inhibitors may be discovered rapidly with modern high-throughput screening and a good biochemical assay, but is often difficult to optimize them for cellular activity. This disconnect between *in vitro* and cellular activities is particularly true for MDR Gram-negative bacteria, where the outer membrane serves as a permeability barrier that limits influx of large, hydrophobic antibiotics into the cell[4]. It is thought that the chemical properties to get in and stay in bacterial cells may be quite different for antibiotics versus molecules typically encountered in pharmaceutical screening libraries[5]. In addition, Gram-negative pathogens possess multidrug efflux pumps, which can reduce the intracellular concentration of antibiotics[6]. Thus, a novel antibiotic requires an aggregate of biochemical potency, good permeability, and desirable efflux properties, all of which must be addressed for bacterial growth inhibition to be observed for drugs that inhibit growth via intracellular targets. To enter the periplasm of Gram-negative bacteria, some biologically-active compounds are thought to transit through protein channels or porins, which favor the passage of small polar molecules[7]. However, the properties required to translocate through porins are at odds with those required to passively diffuse through the inner membrane[5]. The difficulty of meeting these criteria cannot be overstated as a hurdle to the development of novel antibiotics. As well, current economic incentives are not thought to support the development of novel “drugs of last resort” for antibiotic resistance[8]. In light of these challenges, new approaches to aid in understanding essential pathways in Gram-negative bacteria must be explored to aid in the scientific challenges of antibiotic discovery.

**Fig 1:**
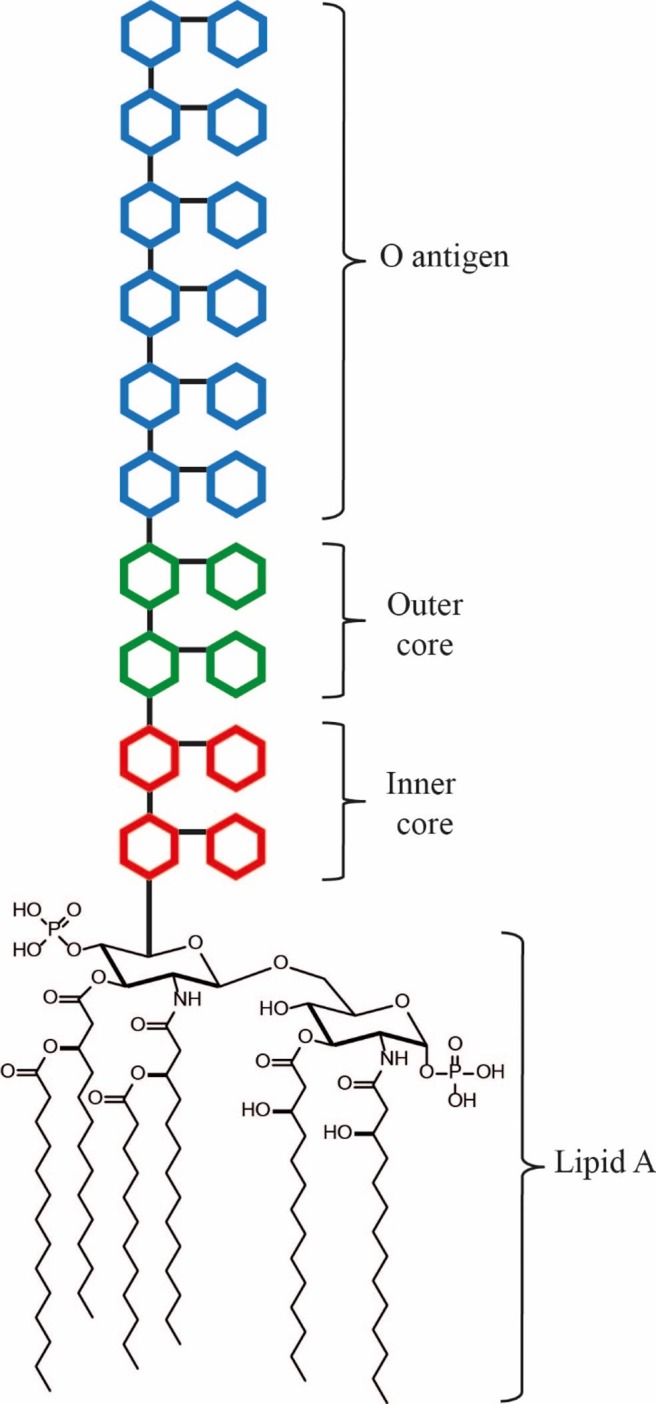
*E*. *coli* Lipid A. *E*. *coli* Lipid A, the lipid moiety of LPS, constitutes the outer leaflet of the outer membrane and anchors LPS to the outer membrane[17].

**Fig 2:**
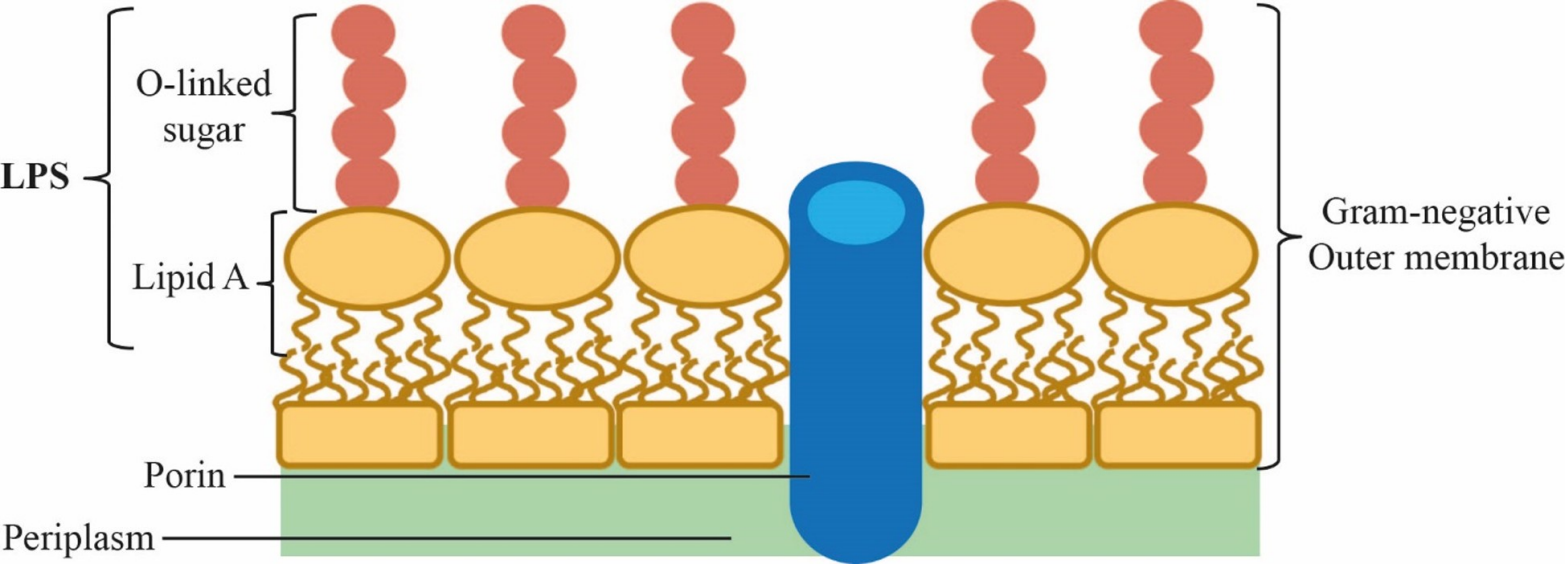
Outer leaflet of Gram-negative bacterial outer membrane. A schematic representation of the Gram-negative bacteria outer membrane, showing Lipid A on the outer leaflet of the outer membrane.

LPS (lipopolysacharride) is a complex glycolipid which is heterogeneous both within and between specific strains of Gram-negative bacteria[9]. LPS consists of lipid A, a variable glycan inner core, a variable glycan outer core, and a variable O-antigen (Fig 1). Lipid A constitutes the outer leaflet of the outer membrane in Gram-negative bacteria and anchors the LPS to the outer membrane (Fig 2). Lipid IV_A_ (7), the product of LpK, represents the last conserved and essential step in the pathway. Lipid IV_A_ (7) is acetylated twice and glycosylated to form Kdo2-Lipid A[10]. By disrupting the LPS biosynthesis pathway, the outer membrane impermeability becomes compromised[11], allowing antibiotics to reach their intracellular targets[12]. Thus, inhibition of Lipid IV_A_ biosynthesis offers the prospect that even small amounts of initial inhibition may facilitate additional uptake due to a self-induced permeability defect. Furthermore, this self-induced permeability defect may also promote the activity of co-administered antibiotics which cannot otherwise cross the outer membrane permeability barrier efficiently[13,14]. Thus enzymes required for Lipid IV_A_ biosynthesis[15,16], such as LpxC, has been considered promising targets for antibiotic discovery. Inhibitors of Lipid IV_A_ biosynthesis can be characterized and optimized by directly monitoring LPS biosynthetic pathway intermediate depletion or accumulation in a cellular context.

LPS is a challenging analyte to monitor due to its exquisite structural diversity and challenges in isolating it[18]. Typical analytical methods of LPS may include the enzymatic LAL assay[19], gels[20,21]_,_ and matrix assisted laser desorption/ionization (MALDI) MS[22]. LPS biosynthetic intermediates are also chemically challenging analytes, due to their detergent like properties which result in poor chromatography. Historically, the Raetz lab and others have labeled LPS and its intermediates with radioactive phosphate or acetate and analyzed them using thin layer chromatography (TLC)[23,24]. Recent developments by the Trent and Brodbelt laboratories applied novel gas-phase fragmentation techniques[25]. In particular, a multiple-reaction monitoring (MRM) LC-MS/MS method was used to quantitate a limited number of LPS biosynthetic intermediates in plants [26]. LC-MS based methods have many advantages compared to infusion MS methods such as the comparison of retention times to authentic standards, reduced ion-suppression, ability to monitor analytes in complex matrices, and improved limits of detection, leading to good qualitative and quantitative performance. Recently, we reported on an MRM reversed phase (RP)-LC-MS/MS method and applications in characterizing an *lptD* mutant strain of *Acinetobacter baumannii*, as well as on an expanded version of this method to analyze LpxH modulation in *A*. *baumannii*[27]^.^ Notably in our previous experiment we included authentic standards for all matching *E*. *coli* metabolites[27]. We also characterized the complex LpxM product *in vitro* by RP-LC-MS/MS[28]. While RP is the standard chromatography method of choice for many analytical chemists, our method for LPS metabolite analysis suffered from several drawbacks including requiring large sample volumes and correspondingly large amounts of compound for treatment to yield detectable signal, high carry-over requiring multiple blanks between samples, and reduced column lifetimes due to backpressure and degradation of resolution. Here we report on a NP-LC-MS/MS method that allows for very sensitive analyte detection, smaller sample volumes, low carry-over, and extended column lifetimes.

**Fig 3:**
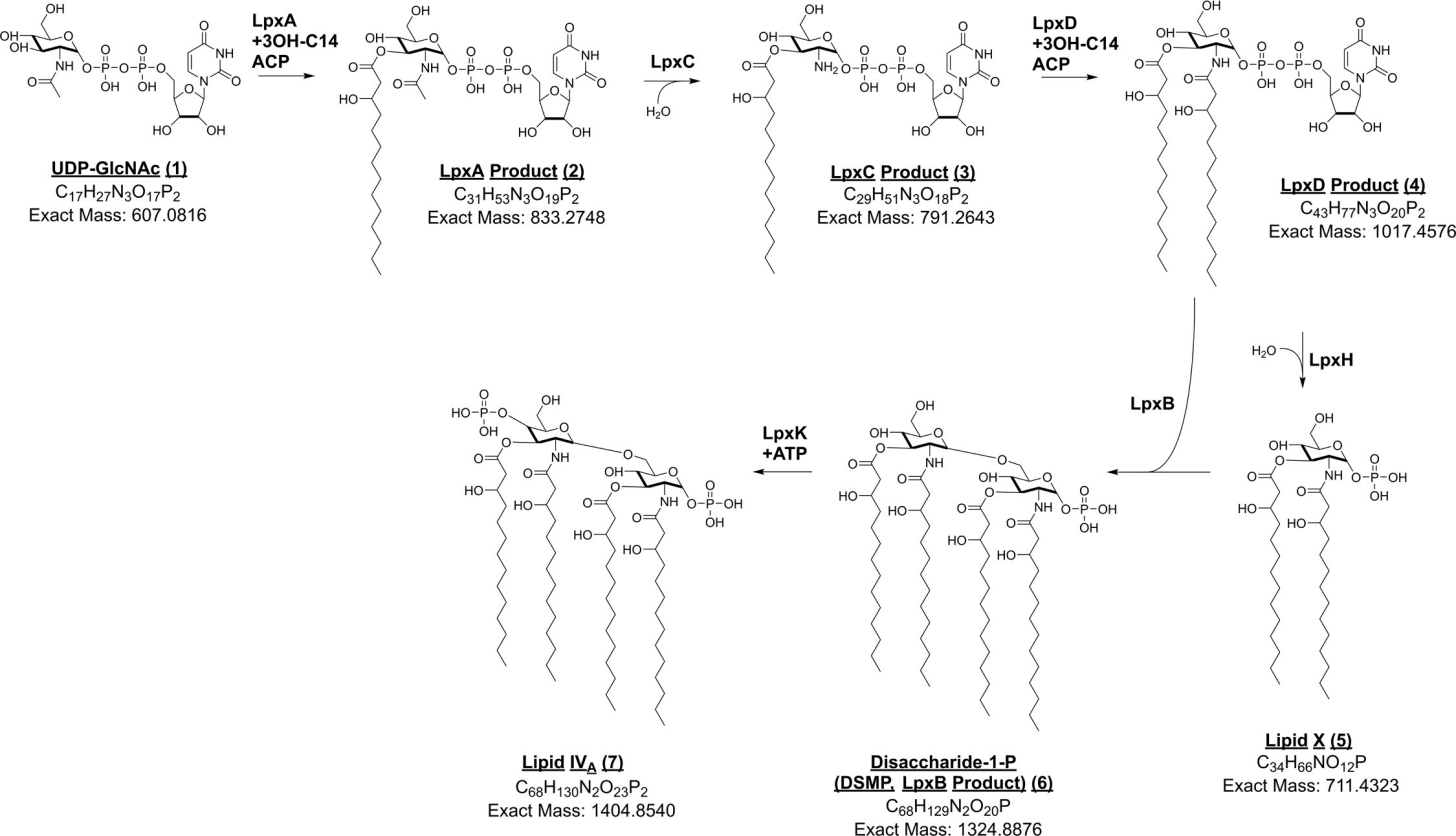
The Raetz pathway for LPS biosynthesis in Gram-negative bacteria. Analytes are shown as monitored with our method for *E*. *coli*. Acyl chain lengths may vary between bacterial species. Molecular formulas and exact mass as depicted are shown. Enzymes are noted for each transformation and analytes are named as both the product of a specific enzyme and/or a common chemical name.

This method to monitor the cellular signal of LPS intermediates of the Raetz pathway (*lpxA*-*lpxK*)[4] in the Gram-negative bacterium *Escherichia coli* (Fig 3) was developed using available and constructed genetic mutants in the Raetz pathway, published dominant acyl variants of the *E*. *coli* LPS intermediates[24], and available tool compounds and standards[11]. Our developed cellular assay consisted of three steps: 1) bacterial cell growth with compound and/or genetic perturbation, 2) lysis and WAX-SPE clean-up, and 3) quantitation by LC-MS. Compound treatment was performed using fold-MIC in order to standardize compound concentration relative to bioactivity. Detergent lysis was used to help solubilize potentially membrane-associated LPS intermediates and to reduce adsorption of the metabolites to plasticware. Waters Oasis mixed-mode WAX-SPE resin allowed enrichment of hydrophobic and phosphate-containing analytes from cell lysates, and removal of interfering molecules that lack an acidic or nonpolar group. This SPE step was also employed for clean-up due to the presence of phosphate groups in all Raetz pathway intermediates of interest. The sensitive and specific technique of multiple reaction monitoring (MRM) LC-MS was then applied to quantitate analytes. Although LC-MS of LPS intermediates has been described in the literature for enzyme assays[29] and cellular studies[26], it has never been a medium-throughput cellular assay. By using normal phase chromatography coupled to mass spectrometry, our assay enables significantly higher throughput than before. We validated our assay procedure in terms of linearity, reproducibility, and sample stability. Furthermore, we showed biological applications in terms of characterizing LPS inhibitors and metabolic profiling of cells treated with antibiotics non-specific to the LPS pathway.

## Materials and methods

### Bacterial strains, plasmids, primers, and growth media

The bacterial strains and plasmids used in this study are listed in Supplementary Tables (S1 and S2 Tables) respectively. All strains used are derivatives of BW25113[30] except for ClearColi K-12 (Lucigen) and deletion alleles were constructed as described previously[31]. A detailed description of construction of the *lpx* controlled-expression strains is given below and in the supplement (S1 and S2 Tables). The ClearColi strain contains mutations that alter LPS structure to reduce endotoxin activity for any derived protein products[32]. The LPS of the ClearColi cell consists largely of lipid IV_A_ after several facile genetic modifications. Thus in our experiments, the ClearColi strain could be used as a control for cells with high amounts of lipid IV_A_. Lysogeny broth (L broth) (tryptone, 10 g/liter; yeast extract, 5 g/liter; NaCl, 10 g/liter) or L agar was used for growth of *E*. *coli* for plasmid and strain construction unless otherwise stated.

### Plasmid construction

All primers used are listed in the supplement (S3 Table). PCR was performed using Phusion High-Fidelity DNA Polymerase (New England BioLabs). Suspension of colonies of the *E*. *coli* wild type strain, BW25113[33] or MG1655 (ATCC 47076), was used as the template. Plasmid DNA and PCR fragments were purified using the Qiaprep spin miniprep kit (Qiagen) or the Qiaquick PCR purification kit (Qiagen), respectively. The *E*. *coli lpxA*, *lpxD*, and *lpxK* genes were PCR-amplified using primers: TU242 and TU243 for *lpxA*, TU115 and TU116 for *lpxD*, and MM18 and MM19 for *lpxK*. The PCR product was digested with EcoRI and HindIII, purified, and cloned into the EcoRI and HindIII sites of pMMB206[34]. Ligation was performed using Quick Ligase (NEB) or Takara Ligation Kit (Takara). DH5α or Top10 competent cells (Thermo Fisher Scientific) were used for transformation followed by selection on L agar supplemented with 10 μg/mL chloramphenicol. The DNA sequences of the inserts were confirmed by nucleotide sequencing by Quintara Biosciences using primers TU40 and TU96. The generated plasmids were designated as pTU457 [pMMB206 P_*lac*_::*lpxA*], pTU433 [pMMB206 P_*lac*_::*lpxD*], and pMM14 [pMMB206 P_*lac*_::*lpxK*].

### Construction of the *lpxA/lpxD/lpxK* conditional expression strains

Due to essentiality of the LPS biosynthesis pathway in *E*. *coli*, the *lpxA*, *lpxD*, and *lpxK* genes could not be entirely knocked out. The native copy of each of the *lpxA*, *lpxD*, and *lpxK* genes was replaced with a kanamycin resistance (Kan^R^) cassette using the λRED system[27] in a strain harboring a plasmid that expresses the corresponding *lpx* gene *in trans*. Briefly, the DNA fragments containing the Kan^R^ cassette with the *lpx* flanking sequences were amplified by PCR using the template pKD13 and primers, TU263 and TU264 for *lpxA*::Kan^R^, TU119 and TU120 for *lpxD*::Kan^R^, and MM20 and MM21 for *lpxK*::Kan^R^. BW25113/pKD46 carrying either of pTU457, pTU433, or pMM14 was grown to late log phase at 30°C in L broth supplemented with 0.2% arabinose, ampicillin (50 μg/mL) and chloramphenicol (10 μg/mL) with shaking. From the culture, the competent cells were made and transformed with the corresponding PCR product using electroporation. Colonies arising on L agar or M9 glycerol minimal agar[35] supplemented with 50 μg/mL kanamycin and 100 μM IPTG were screened for IPTG-dependent growth. The inactivation of the chromosomal copy of *the lpx gene* was confirmed with PCR and sequencing using primers TU276 and TU277 for *lpxA*, TU117 and TU118 for *lpxD*, and MM22 and MM23 for *lpxK*. The resultant *lpxA* controlled-expression strain TUP0005 [*lpxA*::Kan^R^ P_*lac*_::*lpxA*] carried the open reading frame of the kanamycin resistant gene starting from the first codon of *lpxA* and fused to the 18 bases of the 3’ end of the *lpxA* open reading frame. This gene replacement method minimized polar effects of *lpxA*::Kan^R^ on expression of the downstream essential genes in the complex operon. The Km^R^ cassette of the *lpxD*::Kan^R^ was excised using pFLP2 plasmid followed by removal of pFLP2 by sucrose selection. This produced the final *lpxD* controlled-expression strain TUP0001 [*lpxD*::frt P_*lac*_::*lpxD*]. In the *lpxK* controlled-expression strain JWM0004 [*lpxK*::Kan^R^ P_*lac*_::*lpxK*], the kanamycin resistant cassette from pKD13 replaced the genomic copy of *lpxK* similarly to the knockout of the KEIO collection[33].

### Construction of the Δ*tolC* Δ*cdh* strain

The *tolC* gene in the *cdh*::Kan^R^ strain JW3889 was deleted as described above using pKD46 carrying the λRED system[27]. The DNA fragment containing the Cam^R^ cassette with the *tolC* flanking sequences was amplified by PCR using the template pKD3 and primers PT577 and PT578. JW3889[*cdh*::Kan^R^]/pKD46 was transformed with the PCR product and colonies were selected on L agar supplemented with 10 μg/ml chloramphenicol. Following confirmation of *tolC*::Cam^R^ using PCR with primers PT441 and PT435 and erythromycin susceptibility, the Kan^R^ and Cam^R^ cassettes were simultaneously excised by pFLP2. After confirmation of the excision using susceptibility to kanamycin and chloramphenicol and by PCR, pFLP2 was removed by sucrose selection, generating the Δ*cdh* Δ*tolC* strain TUP0035.

### Cell culture for compound-treated cells and ClearColi

*E*. *coli* Δ*cdh* Δ*tolC* were grown overnight (Approximately 18 hours) in cation-adjusted Mueller-Hinton (MHB-II CA) at 37°C in a shaking incubator at 220 rpm. The following day, the culture was diluted to OD_600_ = 0.05 in MHB-II CA and grown for approximately 1 hour at 37°C and 220 rpm to reach OD_600_ = 0.2. After incubation, 1 mL of culture was dispensed into each well of a 96-well deep-well polypropylene plate containing 10 μL of inhibitor. The inhibitors evaluated were CHIR-090 (9), ChemDiv 6359–0284 (10), and ChemDiv C324-2728 (11). In order to standardize compound concentration relative to bioactivity, cells were treated at fold-MICs of compounds. For ClearColi cells, used as tool to measure DSMP and Lipid IV_A_ (7) accumulation, 1 mL of cell culture was dispensed into each well of a 96-well deep-well polypropylene plate with no inhibitors. Plates were then sealed with breathable films and incubated for 1 hour at 37°C and 220 rpm. After incubation, 100 μL was transferred from each well into a 96-well polystyrene plate for OD_600_ reading. To the remaining 900 μL of cell culture, 1 mL of Genlantis SoluLyse detergent containing 1 nM of the C_10_ *Pseudomonas aeruginosa* LpxA product (8) (ARC: Alberta Research Chemicals) as an internal standard was added. Plates were shaken at room temperature for 30 minutes before being spun 3,000 rpm for 15 minutes to pellet cellular debris.

### Cell culture for IPTG-controlled expression strains

The *lpxA*, *lpxD*, and *lpxK* IPTG-controlled expression strains of *E*. *coli* were grown overnight (approximately 18 hours) in MHB-II CA with 100 μM IPTG (Isopropyl β-D-1-thiogalactopyranoside) at 37°C in a shaking incubator at 220 rpm. The following day, the culture was diluted to OD_600_ = 0.05 in MHB-II CA with 100 μM IPTG and incubated at 37°C and 220 rpm until OD_600_ = 0.5. Cells were then pelleted at 10,000 rpm for 10 mins and washed with MHB-II CA three times to remove IPTG before being resuspended in MHB-II CA at OD_600_ = 0.0025 (200X dilution). The resuspended cells were transferred into each well of a 96-well deep-well plate at a volume of 1 mL and then incubated for 3 hours to OD_600_ = 0.2. After incubation, 100 μL was transferred from each well into a 96-well polystyrene plate for OD_600_ reading. To the remaining 900 μL of cell culture, 1 mL of Genlantis SoluLyse detergent containing 1 nM of the C_10_ *Pseudomonas aeruginosa* LpxA product (8) as an internal standard was added. Plates were shaken at room temperature for 30 minutes before being spun 3,000 rpm for 15 minutes to pellet cellular debris.

### Purification of LPS intermediates by SPE extraction

SPE extraction was performed using Waters Oasis WAX SPE 96-well plates (10 mg sorbent per well, 30 μm particle) on a vacuum manifold. SPE was conditioned with 500 μL of methanol and re-equilibrated with 500 μL of water. From the pelleted cell lysate, 2 x 900 μL of supernatant was transferred to SPE, with flow-through being discarded. SPE was washed with 500 μL of 2% formic acid, followed by 500 μL of acetonitrile. LPS intermediates were eluted from SPE with 75 μL of 5% ammonium hydroxide in methanol into a 96-well polypropylene plate containing 15 μL of 10% formic acid in methanol per well to acidify the samples to approximately pH 5–6. Note: allowing the LPS biosynthetic intermediates to incubate in basic solution will result in poor results due to hydrolysis.

### Detection of LPS intermediates by normal phase LC-MS/MS

MRM data was acquired on a Sciex 4000 QTRAP mass spectrometer with Turbo V ion source coupled to an Agilent 1100 LC. Both systems are well-suited to NP operation when the appropriate glass and steel capillaries are used, as well as appropriate pump seals. Other LC-MS systems may be less suitable for the buffers employed Mobile phase A consisted of 80% chloroform, 19.5% methanol, and 0.5% ammonium hydroxide. Mobile phase B consisted of 45% chloroform, 45% methanol, 9.5% water, and 0.5% ammonium hydroxide. LC-MS grade solvents should be employed. Flow was applied at 200 μL/minute to a Waters Acquity UPLC BEH HILIC column, 130 Å pore, 1.7 μm particle, 2.1 × 100 mm. Samples were kept at 4°C in the autosampler prior to injecting 10 μL onto the column. Samples were loaded in 10% mobile phase B and this composition was held for the first minute of chromatography. Mobile phase B was increased linearly to 70% over 3 minutes and to 99% at 6 minutes, held at 99% until 9 minutes, and then reduced to 10% at 9.1 minutes. The system was re-equilibrated for 4 minutes between sample injections (total 13.1 minutes per sample run). MS/MS data was collected between minutes 2–11. The mass spectrometer source heater was set at 450°C, IonSpray voltage = -4500 V, curtain gas = 10 L/min, collision gas = 8 L/min, nebulizer gas = 35 L/min, heater gas = 40 L/min. MRM settings are shown in Table 1. MRM data were acquired as shown in Table 1 with a duty cycle of 1.26 seconds per scan. All transitions were tuned on samples of authentic standards in infusion mode prepared as described[27–28,33]. Integrated retention times were matched to authentic standards elution profiles as well. Note: care should be taken to ensure that the mass spectrometer waste gas is appropriately vented out of the laboratory due to the health risks associated with normal phase solvents. Consult vendor manuals for correct installation of waste lines.

**Table 1:** MRM settings for monitoring *E*. *coli* LPS intermediates and *P*. *aeruginosa* internal standard.

Analyte	Q1 (*m/z*)	Q1 (res)	Q3 (*m/z*)	Q3 (res)	Dwell (ms)	DP (V)	EP (V)	CE (V)	CXP (V)
*E*. *coli* LpxA product (2)	832.3	Unit	158.9	Unit	40	-100	-10	-80	-12
*E*. *coli* LpxA product (2)	832.3	Unit	273	Unit	40	-100	-10	-70	-10
*E*. *coli* LpxA product (2)	832.3	Unit	385	Unit	40	-100	-10	-50	-10
*E*. *coli* LpxC Product (3)	790.3	Unit	158.9	Unit	40	-90	-10	-85	-12
*E*. *coli* LpxC Product (3)	790.3	Unit	273	Unit	40	-90	-10	-65	-10
*E*. *coli* LpxC Product (3)	790.3	Unit	385	Unit	40	-90	-10	-52	-10
*E*. *coli* LpxC Product (3)	790.3	Unit	546.2	Unit	40	-90	-10	-44	-5
*E*. *coli* LpxD Product (4)	1016.5	Unit	158.9	Unit	40	-120	-10	-108	-10
*E*. *coli* LpxD Product (4)	1016.5	Unit	273	Unit	40	-120	-10	-74	-10
*E*. *coli* LpxD Product (4)	1016.5	Unit	385	Unit	40	-120	-10	-60	-10
*E*. *coli* Lipid X (5)	710.4	Unit	466.2	Unit	40	-80	-10	-50	-10
*E*. *coli* Lipid X (5)	710.4	Unit	240.1	Unit	40	-80	-10	-73	-10
*E*. *coli* DSMP (6)	1323.9	Unit	79	Unit	40	-120	-10	-130	-10
*E*. *coli* DSMP (6)	1323.9	Unit	1079.7	Unit	40	-120	-10	-90	-12
*E*. *coli* DSMP (6)	1323.9	Unit	835.5	Unit	40	-120	-10	-80	-5
*E*. *coli* DSMP (6)	1323.9	Unit	895.5	Unit	40	-120	-10	-80	-5
*E*. *coli* DSMP (6)	1323.9	Unit	651.3	Unit	40	-120	-10	-110	-5
*E*. *coli* Lipid IV_A_ (7)	701.4	Unit	243.2	Unit	40	-110	-10	-45	-15
*E*. *coli* Lipid IV_A_ (7)	701.4	Unit	79	Unit	40	-110	-10	-130	-5
*E*. *coli* Lipid IV_A_ (7)	701.4	Unit	588.3	Unit	40	-110	-10	-35	-15
*E*. *coli* Lipid IV_A_ (7)	701.4	Unit	466.2	Unit	40	-110	-10	-40	-15
*E*. *coli* Lipid IV_A_ (7)	701.4	Unit	1079.7	Unit	40	-110	-10	-35	-15
UDP-GlcNAc (1)	606.4	Unit	385	Unit	40	-80	-10	-37	-5
UDP-GlcNAc (1)	606.4	Unit	282.4	Unit	40	-98	-10	-42	-11
UDP-GlcNAc (1)	606.4	Unit	273	Unit	40	-91	-10	-46	-14
*P*. *aeruginosa* LpxA product (8)	776.3	Unit	158.9	Unit	40	-118	-10	-80	-5
*P*. *aeruginosa* LpxA product (8)	776.3	Unit	273	Unit	40	-118	-10	-60	-5
*P*. *aeruginosa* LpxA product (8)	776.3	Unit	385	Unit	40	-118	-10	-45	-5

### Data handling

Data were acquired on an AB Sciex Analyst workstation (v1.6.2, build 8489). Once peaks had been integrated, transitions for each parent mass were summed. Total parent *m/z* peak area was normalized to OD_600_ and the peak area of the C10 *P*. *aeruginosa* LpxA product (8) internal standard. Normalization to OD_600_ was done to account for differences in total cell mass between samples. Normalization to peak area of the internal standard was done in order to account for well to well variability as well as for any variations in injection volume by the autosampler.

## Results and discussion

### Method optimization

#### Optimization of chromatography and acquisition

The separation and retention of the LPS intermediates was evaluated by the normal phase (NP) method above and reversed-phase (RP) chromatography[33, 29]. Despite achieving good separation by the RP method, this approach was deprioritized due to high amounts of carry-over between samples. Among NP columns assessed, the Waters Acquity UPLC BEH HILIC column yielded the best separation and peak shape of the LPS intermediates and was stable under the basic mobile phase conditions used. Other NP columns tested were 1) YMC Pack PVA-Sil, 5 μm particle, 120 Å pore, 2.1 × 100 mm, 2) Phenomenex Luna NH2, 3 μm particle, 100 Å pore, 2.1 × 30 mm, and 3) Ascentis Express HILIC, 5 μm particle, 2.1 × 50 mm. MRM transitions were determined and optimized previously in the literature [27].

#### Optimization of sample preparation

Due to the complex nature of cellular lysate, an SPE step greatly increased signal of LPS intermediates by reducing signal from interfering matrix and concentrating desired analytes. Furthermore, injecting cellular lysate samples without a clean-up step regularly resulted in clogging of the HPLC system and the column. SPE development was performed on a Waters Oasis Method Development 96-well μElution plate, containing mixed-mode strong anion-exchange (MAX), mixed-mode strong cation-exchange (MCX), mixed-mode weak anion-exchange (WAX), or mixed-mode strong cation-exchange (WCX) resins. Additionally, Waters Oasis hydrophilic-lipophilic balance (HLB) resin was also tested. Standards of the Raetz pathway intermediates were diluted in *P*. *aeruginosa lysate* before processing by SPE. Lysate from *P*. *aeruginosa* was used to mimic the *E*. *coli* sample matrix with the noted difference in masses because the LPS intermediates in this bacterium have a different acyl chain lengths compared to *E*. *coli*. The mixed-mode WAX resin performed best for isolating the LPS intermediates of all the resins tested, presumably due to the hydrophobic and negatively-charged nature of these analytes.

#### Enhancement of early pathway LPS intermediate signals by CDH deletion

**Fig 4:**
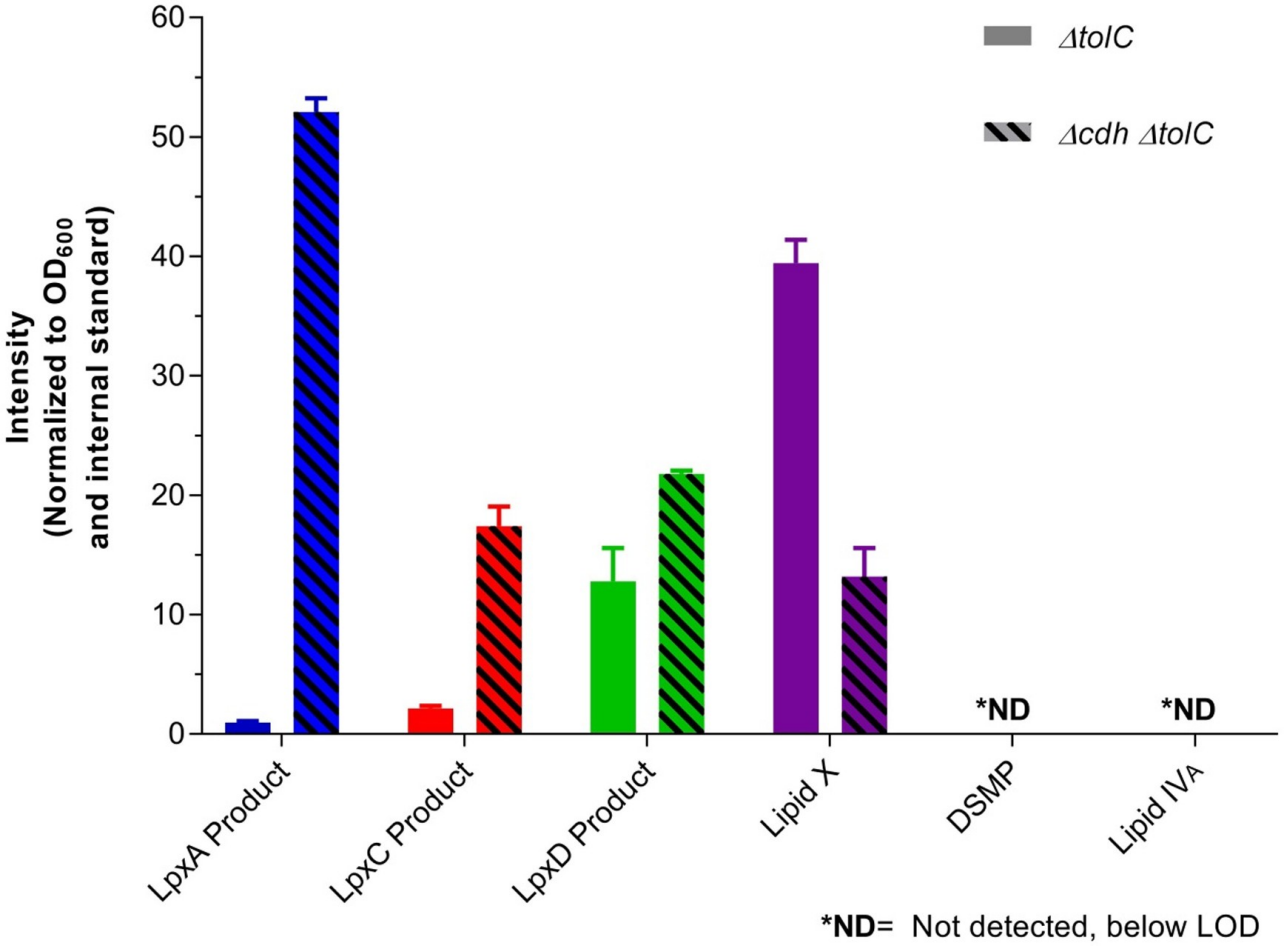
Comparison of LPS intermediates between *E*. *coli ΔtolC* and *E*. *coli Δcdh ΔtolC*. CDH-diacylglycerol pyrophosphatase in *E*. *coli ΔtolC* remains active in detergent lysis conditions, as indicated by increased signal of LpxA product (2), LpxC product (3), and LpxD product (4) as well as decreased signal of Lipid X (5) in *E*. *coli Δcdh ΔtolC*.

Membrane-associated CDH-diacylglycerol pyrophosphatase, encoded by the *cdh* gene in *E*. *coli*, hydrolyzes CDP-diacylglycerol into cytidine monophosphate (CMP) and phosphatidic acid in the periplasm. Upon cell lysis, CDH-diacylglycerol pyrophosphatase can access the LpxD product (4, UDP-diacyl-GlcN) located in the cytoplasm and hydrolyze it in a similar manner to LpxH, yielding an increase in Lipid X in assay readout (5) [36]. Because SoluLyse is a non-denaturing detergent lysis, we tested if post-lysis CDH activity was confounding the assay. LPS intermediates were extracted and quantified from *E*. *coli ΔtolC* and *E*. *coli Δcdh ΔtolC*, and normalized signal was compared between the two (Fig 4 and S4 Table). DSMP (6) and Lipid IV_A_ (7) levels were below limit of detection. An increase of intermediates upstream of LpxH and decrease of Lipid X (5) (LpxH product) were observed in the *E*. *coli Δcdh ΔtolC* cells, indicating that CDH-diacylglycerol pyrophosphatase activity is preserved after lysis and affects results of early pathway intermediates (Fig 4). Thus by performing our assay in *Δcdh* cells, analyte signal could be increased. To avoid hydrolysis by CDH in the lysates from the *ΔtolC* cells, alternative denaturing lysis methods such as organic solvent were explored, however, none yielded better signal of the analytes or abolished the pyrophosphatase activity of CDH-diacylglycerol pyrophosphatase.

#### Growth condition for metabolite analysis of compound-treated *E*. *coli* cells

**Fig 5:**
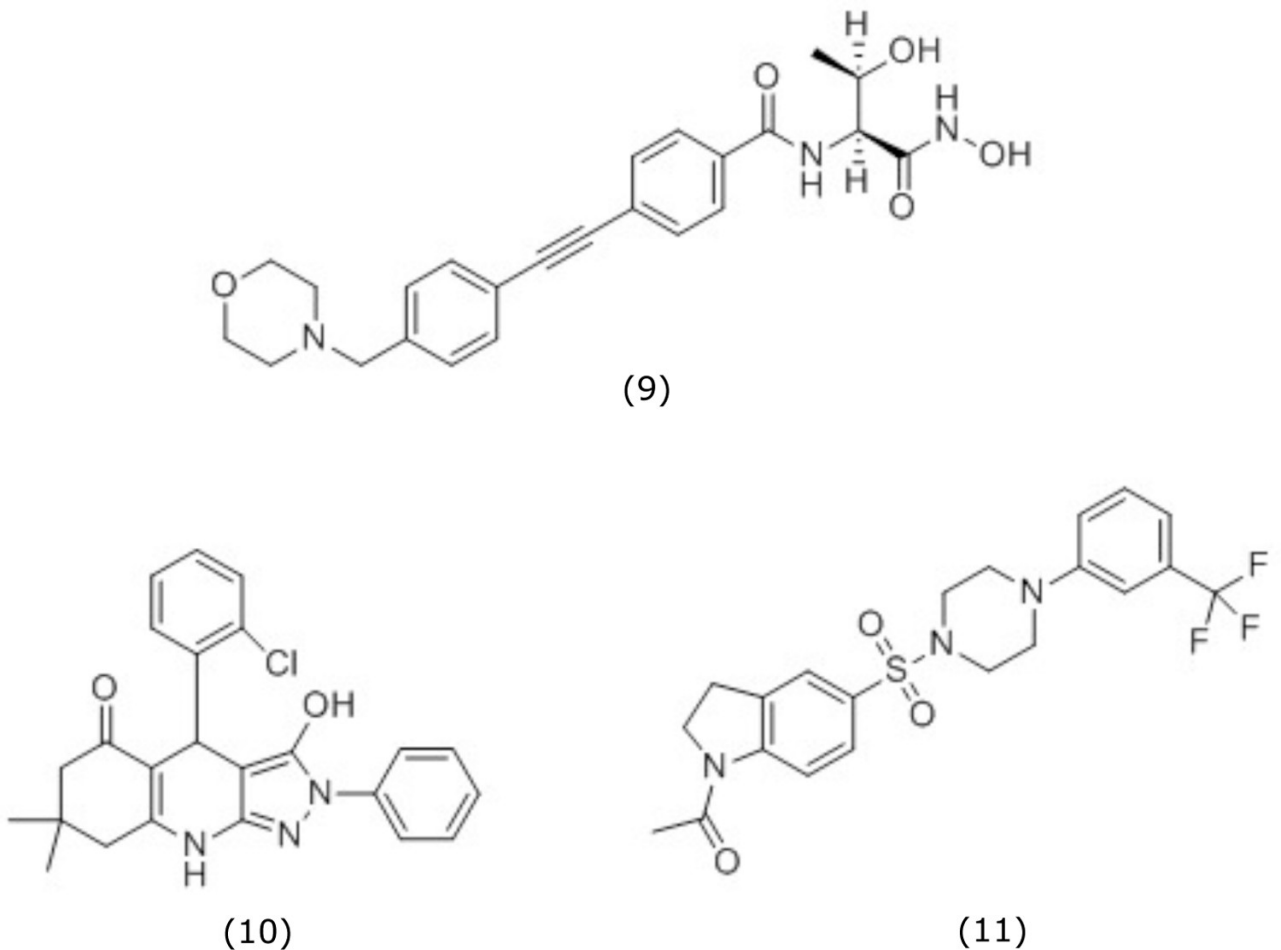
Compound structures of tested LPS inhibitors. CHIR-090 (9), ChemDiv 6359–0284 (10), and ChemDiv C324-2728 (11)9. CHIR-090 (9) is a LpxC inhibitor[15]_,_ ChemDiv 6359–0284 (10) is a LpxD inhibitor[17], and ChemDiv C324-2728 (11) is a LpxH inhibitor[16].

**Fig 6:**
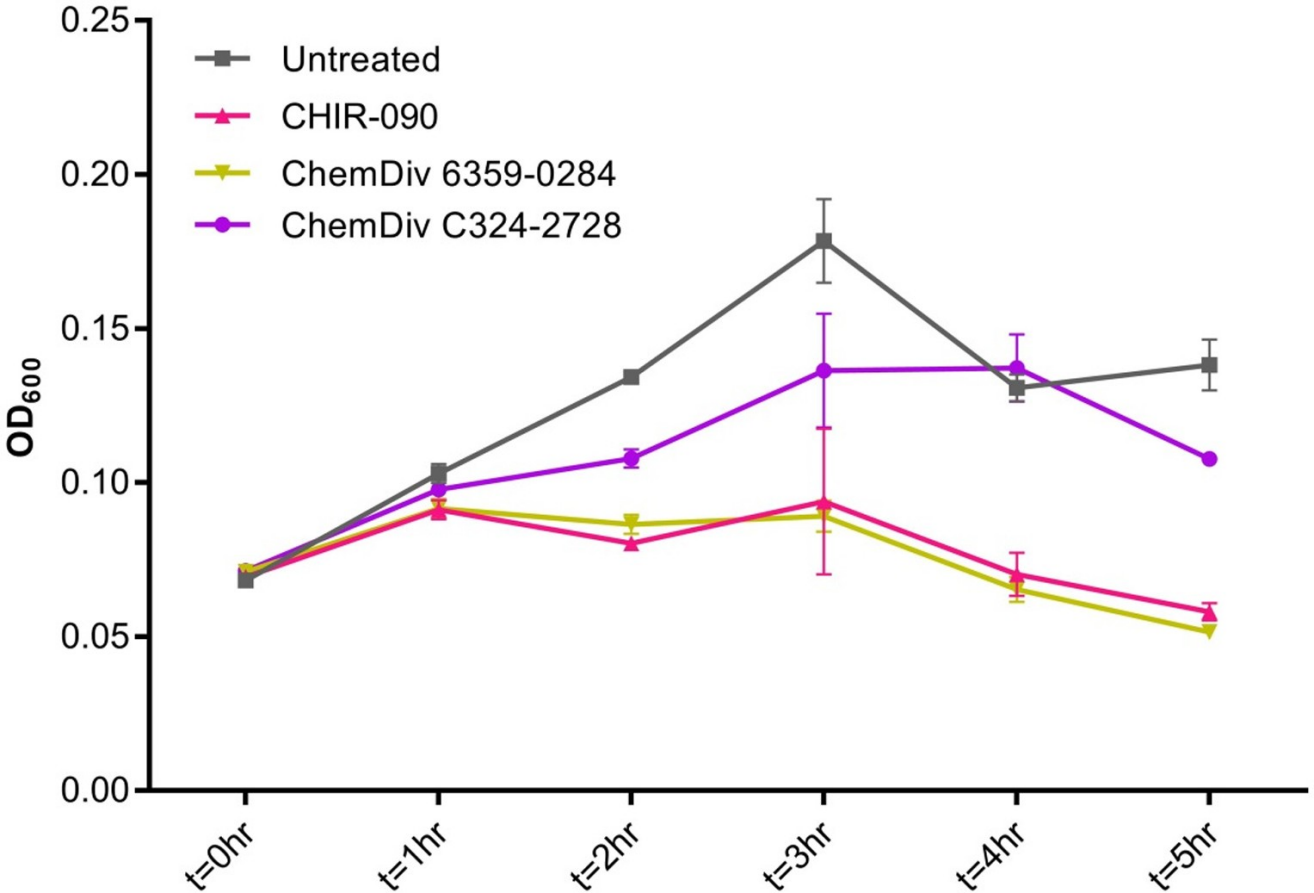
OD_600_ values for untreated and compound treated cells. OD_600_ values for cells treated at 8X MIC with LpxC inhibitor (CHIR-090 (9), 0.032 μg/mL) and putative LpxH inhibitor (ChemDiv C324-2728 (11), 16 μg/mL) show little difference compared to untreated cells at 1 hour; However treatments longer than 1 hour show very different OD_600_ values for the three conditions, indicating that cell density and viability is not consistent between conditions at these time-points.

To determine whether the substrate of an enzyme increased with inhibition by corresponding inhibitors, *ΔtolC Δcdh* cells treated with inhibitors (Fig 5) at 4X MIC and grown for a total of 6 hours. The 4X MIC concentration was used in order to ensure quantifiable changes in the intermediates. Cultures were sampled hourly (n = 3) to determine differences in cell density as a function of time (Fig 6). Due to the bioactivity of the probe compounds, we chose a 1 hour time-point for harvesting cells after compound treatment to minimize differences in OD_600_ readings between untreated and treated cells. The ideal time-point for the assay, which may differ between compound and mechanism of action, would be late enough to observe a significant phenotype in the targeted pathway such as LPS metabolite perturbation, but not so late as to lose metabolites into media by the specific phenotype, for example, with lysis by LpxC inhibitors. For measuring LPS metabolites in compound-treated cells, OD_600_ was used as a proxy for cell lysis and growth inhibition so the ideal time-point could be selected.

#### Changes in LPS intermediate levels by genetic and chemical perturbations

**Fig 7:**
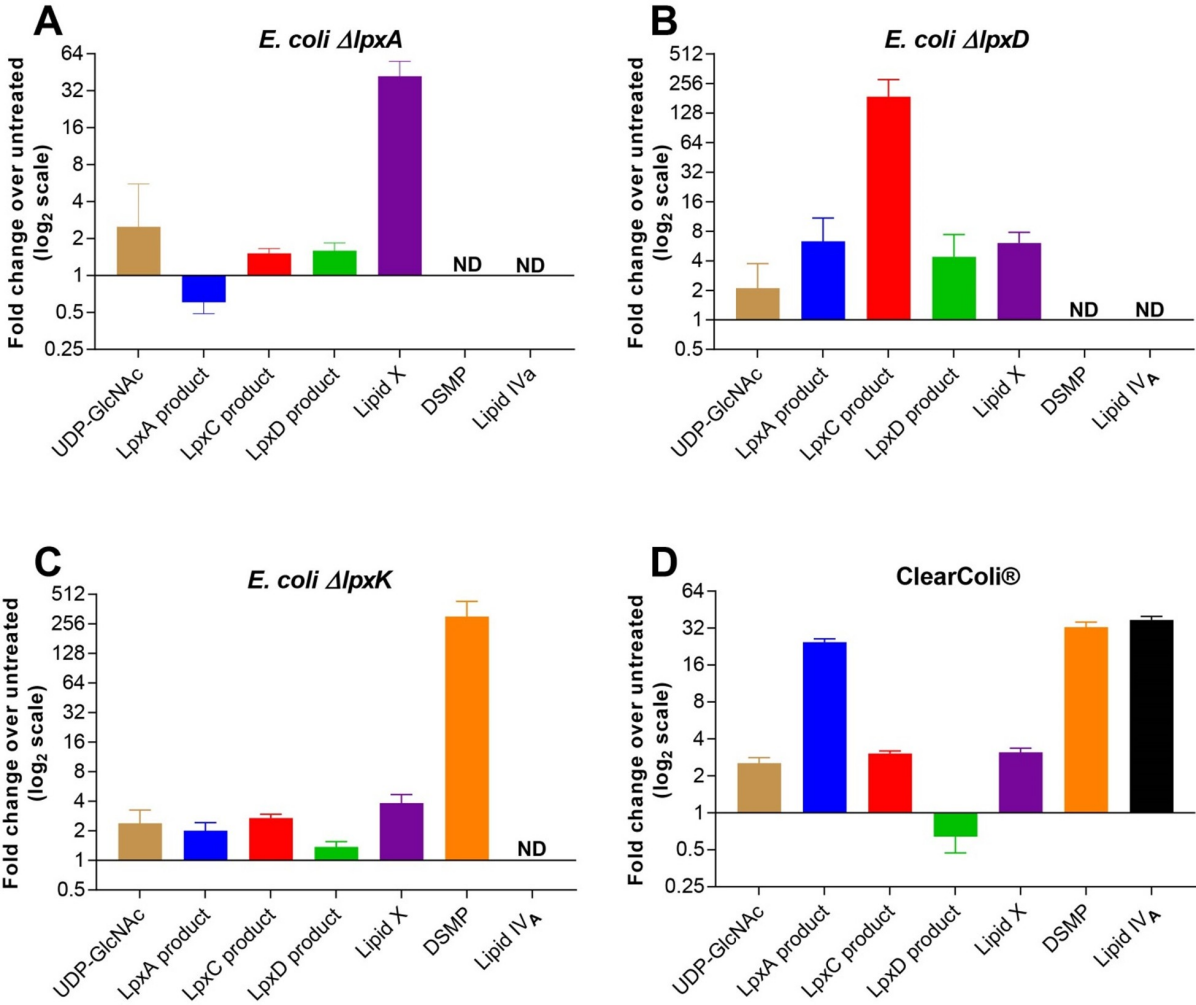
Relative levels of LPS intermediates in LpxA/D/K-depleted cells measured by NP-LCMS/MS. Peak areas of each transition were summed for each parent mass and normalized against OD_600_ and the C10 *Pseudomonas aeruginosa* LpxA product (8) internal standard. Normalized responses of genetically perturbed cells were then compared against untreated *E*. *coli ΔtolC* cells. Metabolites with signal below the limit of detection are denoted by ND. (A) Fold change of LPS intermediates in LpxA depletion (n = 3) for 3 hours compared to untreated *E*. *coli ΔtolC* cells. (B) Fold change of LPS intermediates after LpxD depletion (n = 3) compared to untreated *E*. *coli ΔtolC* cells. (C) Fold change of LPS intermediates after LpxK depletion (n = 3) compared to untreated *E*. *coli ΔtolC* cells. (D) Fold change of LPS intermediates in ClearColi cells (n = 3) compared to untreated *E*. *coli ΔtolC* cells. Error bars represent the standard deviation of the fold change of treated cells over untreated cells.

LPS pathway metabolites were investigated by utilizing tool inhibitors of the pathway, controlled-expression strains (LpxA, LpxD, and LpxK), and the ClearColi strain. ClearColi has been genetically modified not to biosynthesize hexa-acylated Kdo_2_-lipid A, the final product of the Raetz pathway[37], by deleting *lpxL*, *lpxM*, *gutQ*, and *kdsD* as well as suppressor mutations to support growth with the loss of two acyl chains and Kdo. These deletions in the ClearColi strain terminated LPS biosynthesis with LpxK being the final step, resulting in much higher levels of DSMP (6) and Lipid IV_A_ (7). All samples were prepared as described in the methods section. In the untreated cells, LpxD product (4) and Lipid X (5) had the highest signal. DSMP (6) and Lipid IV_A_ (7) were below the limit of detection without perturbing the pathway to cause their accumulation. LpxA product (2) has low signal in the untreated cell and therefore the depletion caused by the genetic perturbation was difficult to quantitate, as the signals rapidly approach the limit of detection. However, the LpxA genetic depletion strain yielded a 2-fold decrease in LpxA product (2) compared to untreated cells (Fig 7A). Given that UDP-GlcNAc (1), the LpxA substrate, can be funneled into other pathways, such as peptidoglycan synthesis, the absence of a large accumulation can be rationalized. Unexpectedly, LpxA depletion via genetic perturbation resulted in a 32-fold accumulation of Lipid X (5). Because LpxA and LpxD share the same β-hydroxymyristoyl-ACP substrate, and its intracellular concentration has been reported to be very low[32], we can hypothesize that as LpxA activity in the cell decreases over time, more β-hydroxymyristoyl-ACP becomes available for LpxD. The LpxD product (4) would be diverted rapidly to Lipid X (5), resulting in its accumulation[38]. This may present a possible explanation for why the accumulation of Lipid X (5) was observed when depleting LpxA.

LpxD genetic depletion resulted in 187-fold increase of its substrate, the LpxC Product (3), and minor accumulations of intermediates further upstream (Fig 7B). However, the LpxD Product (4) and Lipid X (5) both had approximately a 4-fold increase. This increase may suggest some turnover of the LpxD enzyme despite the depletion. Since the substrate had accumulated to such a high level, this could push the reaction forward, slightly increasing production of the downstream metabolites.

The LpxK depletion strain was used to understand the effect by LpxK inhibition on the pathway since currently there is no tool inhibitor molecule reported. As expected, LpxK depletion resulted in a 256-fold increase of its substrate, DSMP (6), with moderate increases in other upstream LPS intermediates, indicating possible product inhibition of upstream enzymes (Fig 7C).

The deletions of *lpxL* and *lpxM* in ClearColi caused an increase in the intermediates except for LpxD product (4) (Fig 7D). This decrease of LpxD product (4) may be a result of two steps: 1) the accumulation of DSMP (6) inhibiting the LpxB activity causing an increase in LpxD Product (4) and Lipid X (5), and 2) LpxH converting accumulated LpxD Product (4) to Lipid X (5). Alternatively, ClearColi may have high CDH activity that converts LpxD product (4) to Lipid X. Interestingly, more minor changes are seen to further upstream intermediates. These metabolic changes may be due to the energetically favorable hydrolysis of UDP from the upstream intermediates such as LpxD product (4).

**Fig 8:**
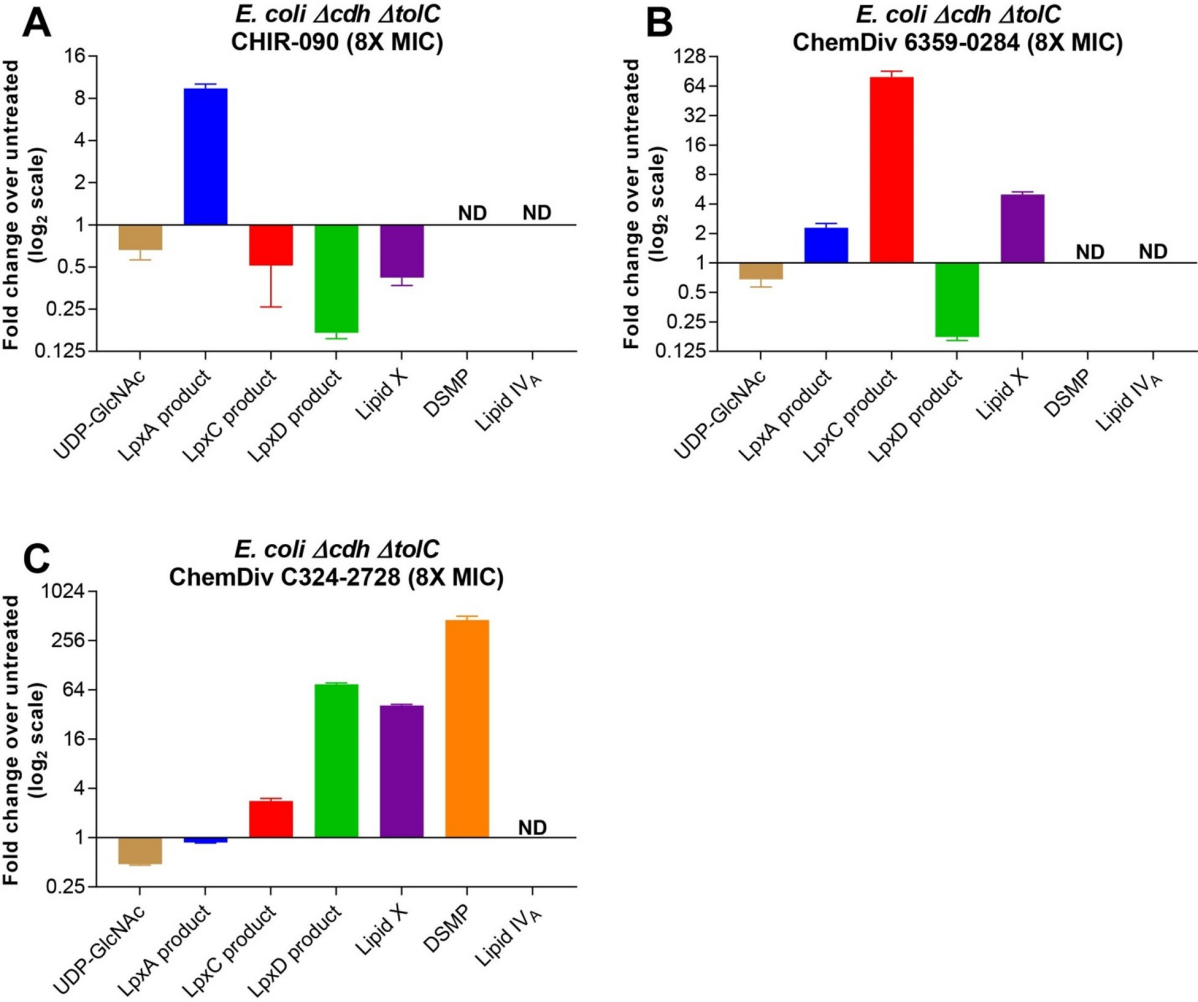
Relative levels of LPS intermediates in genetically depleted cells measured by NP-LCMS/MS. (A) Fold change of LPS intermediates in *E*. *coli Δcdh ΔtolC* cells treated with CHIR-090 (9) (n = 8) at 8X MIC compared to untreated *E*. *coli Δcdh ΔtolC* cells. (B) Fold change of LPS intermediates in *E*. *coli Δcdh ΔtolC* cells treated with ChemDiv 6359–0284 (10) (n = 8), an LpxD inhibitor, at 8X MIC compared to untreated *E*. *coli Δcdh ΔtolC* cells. (C) Fold change of LPS intermediates in *E*. *coli Δcdh ΔtolC* cells treated with ChemDiv C324-2728 (11) (n = 8), a putative LpxH inhibitor, at 8X MIC compared to untreated *E*. *coli Δcdh ΔtolC* cells. Error bars represent the standard deviation of the fold change of treated cells over untreated cells.

Treatment of *E*. *coli Δcdh ΔtolC* cells with CHIR-090 (9) at 8X MIC (0.032 μg/mL) caused a 10-fold accumulation of its substrate, the LpxA Product (2) (Fig 8A). The 8X MIC concentration was used since that was the concentration at which the changes in levels of LPS intermediates began to plateau for the tool molecules used in this experiment. Additionally, the 8X MIC concentration did not produce significant reduction in the OD_600_ of the treated culture compared to the untreated culture after 1 hour incubation (Fig 6). Reduction of the LpxC Product (3) was difficult to quantify, since LpxC Product (3) has low signal in the untreated cell and treatment with the CHIR-090 (9) caused its signal to fall below the limit of detection. LpxD Product (4) and Lipid X (5), which have sufficient signal to robustly report on, showed a 4-8-fold reduction due to chemical inhibition of LpxC as expected.

Treatment of cells with ChemDiv 6359–0284 (10) at 8X MIC (16 μg/mL) caused a 64-fold accumulation of LpxC Product (3) and a 16-fold decrease in LpxD Product (4) (Fig 8B), consistent with LpxD inhibition. A moderate increase in upstream LpxA product (2) was observed, as well as a 4X increase in downstream Lipid X (5). This increase of Lipid X (5) is presumably due to the little remaining LpxD Product (4) being diverted to Lipid X (5) instead of forming DSMP (6).

Treatment of cells with the LpxH inhibitor, ChemDiv C324-2728 (11), at 8X MIC (2 μg/mL) resulted in a 64-fold increase in its substrate the LpxD Product (4). However, treatment also yielded an unexpected 32-fold increase in Lipid X (5), the product of LpxH, and a 64-fold increase in DSMP (6) further downstream (Fig 8C). Although previously-hypothesized metabolic channeling by LpxH and LpxB[12] might explain this accumulation of downstream products in the presence of the LpxH inhibitor, understanding of how this inhibitor metabolically interacts with this step of the pathway would require further investigation to fully understand how this inhibitor interacts with this step of the pathway. An *lpxH*-controlled expression strain was not available to examine whether the similar metabolic changes are observed upon LpxH depletion.

#### Evaluation of assay robustness

The method robustness was evaluated by preparing *E*. *coli Δcdh ΔtolC* treated with 3 tool molecules (CHIR-090 (9), ChemDiv 6359–0284 (10), and ChemDiv C324-2728 (11)), as well as an untreated control. The ClearColi *E*. *coli* strain was also evaluated since it accumulates Lipid IV_A_ (7)[13]. Compounds were tested at 8X MIC (n = 8) and these controls were carried out over three different days. Reduction of DSMP (6) and Lipid IV_A_ (7) was not measured since inhibition of their production lowered the signal for these intermediates below the limit of detection. Signals for treated and untreated cells were normalized to OD_600_ and the *P*. *aeruginosa* LpxA product (8) internal standard, and fold-changes for each condition were calculated by dividing the normalized signal by the normalized signal of the untreated cells. Average fold changes across days (Table 2) and the corresponding %CV (Table 3) were calculated. Levels of accumulation were consistent with experiments done outside of method validation.

**Table 2:** Average accumulation of LPS intermediates in three independent experiments with compounds at 8X MIC (n = 8).

	LpxA product (2)	LpxC Product (3)	LpxD Product (4)	Lipid X (5)	DSMP (6)
Treatment	CHIR-090 (9)	ChemDiv C324-2728 (11)	ChemDiv C324-2728 (11)	ChemDiv C324-2728 (11)	ChemDiv C324-2728 (11)
Average fold change over untreated cells	10	83	106	20	75

**Table 3:** %CV of fold change across independent experiments for compound-treated *E*. *coli Δcdh ΔtolC*.

	%CV						
Treatment	UDP-GlcNAc (1)	LpxA product (2)	LpxC Product (3)	LpxD Product (4)	Lipid X (5)	DSMP (6)	Lipid IV_A_ (7)
CHIR-090 (9)	12	7	16	12	13	-	-
ChemDiv 6359–0284 (10)	15	11	25	12	5	-	-
ChemDiv C324-2728 (11)	19	18	23	17	25	13	15
ClearColi	4	13	20	-	21	6	1

%CV of fold-change for metabolites UDP-GlcNAc (1), LpxA, LpxC, LpxD products (2, 3, 4), and Lipid X (5) were <25% for compound treated conditions. DSMP (6) and Lipid IV_A_ (7) had high variability due to their abundance being below the limit of detection in conditions where they do not accumulate. In conditions where DSMP (6) and Lipid IV_A_ (7) accumulate, such as with ChemDiv C324-2728 (11) treatment and ClearColi cells, %CV of fold-change for these intermediates were <15%. ClearColi cells showed a decrease of the LpxD Product (4) where the signal was below the calculated limit of detection, therefore the %CV was not calculated.

### Responses in LPS metabolites to antibiotic treatment

**Fig 9:**
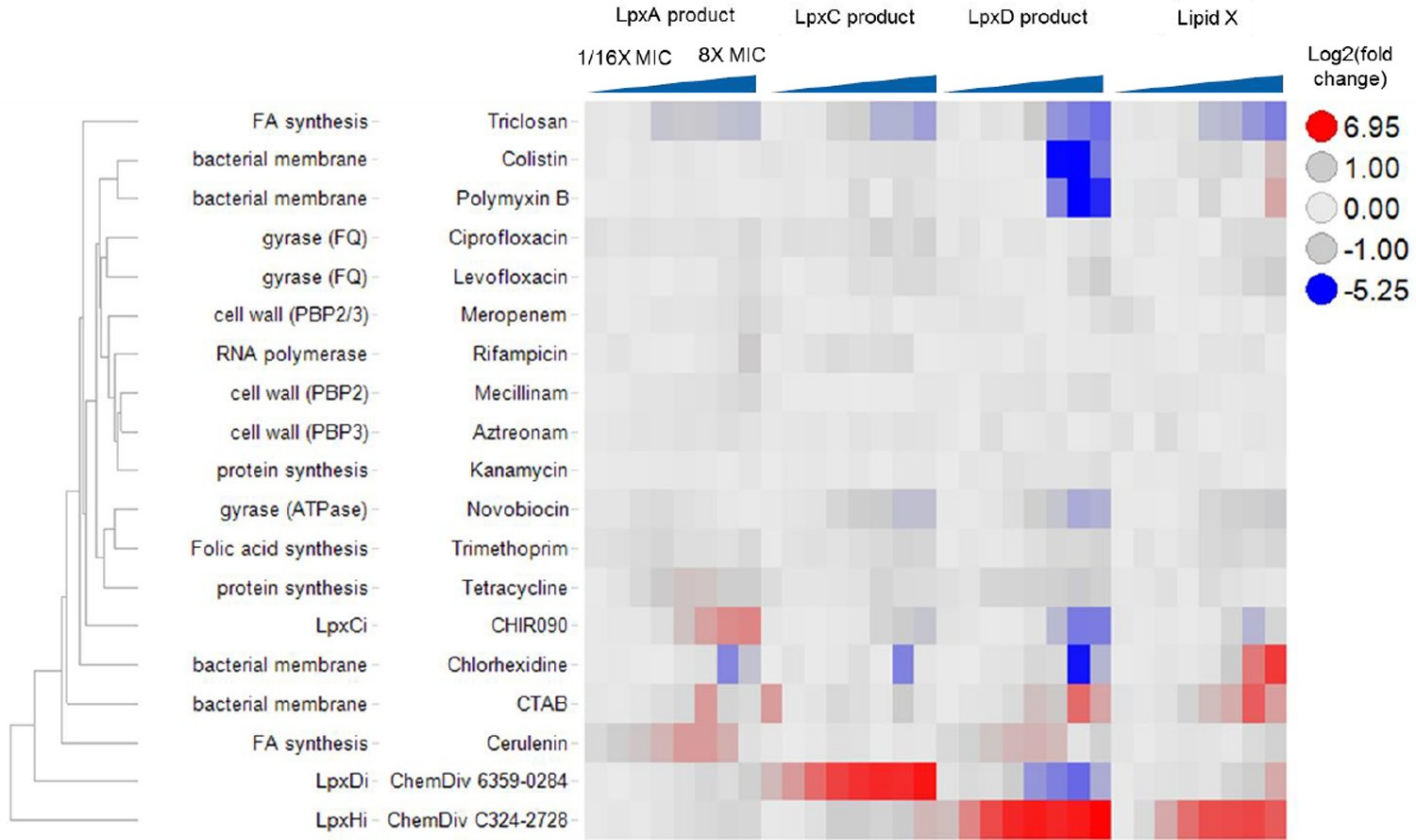
Heatmap displaying LPS intermediate levels of *E*. *coli Δcdh ΔtolC* cells treated with various antibiotics in dose-response. The red color indicates an accumulation, grey indicates no significant change, and blue indicates depletion. Hierarchical clustering was performed in Spotfire (version 6.5.3) and displayed as a row dendogram. Values in all columns were normalized to scale between 0 and 1 and used in the clustering calculation. Distances between all possible combinations of two rows were calculated using Euclidean distance measure. These calculated distances were then used in the UPGMA clustering method to derive the distance between all clusters that were formed from the rows during the clustering.

To better understand nonspecific perturbations of the pathway, *E*. *coli Δcdh ΔtolC* was treated with a panel of antibiotics with varying mechanisms of action. All antibiotics were tested in two-fold dilution series starting from 8X MIC to 1/16X MIC and repeated over two different days. Log2 of the fold changes for LpxA product (2), LpxC Product (3), LpxD Product (4), and Lipid X (5) at each concentration of the antibiotic were determined and are displayed as a heat map (Fig 9). DSMP (6) and Lipid IV_a_ were excluded from the heat map due to high variability in the data since they could not be detected in most cases. From the assay reproducibility experiment, fold changes between 2 and 0.5 (log2 scale -1 to 1) were within the variability of the assay and are colored in grey. Fold changes greater than 2 (log2 scale > 1) indicate significant accumulation and are colored in red. Fold changes less than 0.5 (log2 scale < -1) indicate significant depletion and are colored in blue. Based on the data, hierarchical clustering of antibiotics was determined (Fig 9).

ChemDiv C324-2728 (11) showed the most divergent profile since it accumulated LpxD Product (4), Lipid X (5), and DSMP (6). The LpxD inhibitor ChemDiv 6359–0284 (10) was the next most divergent since it was the only compound that showed accumulation of the LpxC Product (3) and a simultaneous depletion of the LpxD Product (4). Cerulenin showed accumulation of both the LpxA Product (2) and LpxD Product (4). Because fatty acid and LPS synthesis are interconnected, these accumulations could be a result of changes in levels of fatty acid substrates. Frank membrane disruptors like CTAB and chlorhexidine showed accumulations of metabolites at specific concentrations, especially for DSMP (6) and Lipid IV_a_ (not shown). This observed increase in DMSP and Lipid IV_A_ (7) could be due to the membrane disruptors aiding in the release of these metabolites, which are membrane bound. The LpxC inhibitor showed an accumulation of its substrate LpxA product (2) and a simultaneous depletion of the LpxD Product (4). In the middle of the heat map are inhibitors of cell wall, protein, and gyrase; these antibiotics did not have significant effect on the LPS metabolites. At the top of the heat map are a fatty acid inhibitor triclosan and bacterial membrane disruptors colistin and polymyxin B that were grouped close together. These compounds all showed a strong decrease of LpxD Product (4). The test compounds that inhibit pathways other than the lipid A biosynthesis pathway decreased LPS metabolites, suggesting that a) depletion is a less specific phenotype or b) substantial metabolic cross-talk exists between the pathways noted. This heat map is a preliminary visualization of the relationships among all the compounds tested and more quantitative approaches to classify the phenotypes are still under development.

## Conclusion

Here we present a robust method for monitoring the lipid IV_A_ biosynthesis pathway and quantitative evaluation of inhibitors that target this pathway. At the time point examined in this assay, the concentration of these pathway intermediates varied depending on the step of the pathway, as indicated by our ability to detect certain analytes better than others despite similar limits of detection (S2 Fig). Metabolic profiling of chemical and genetic perturbations of the early LPS biosynthetic pathway revealed accumulation of substrates in response to chemical inhibition or genetic perturbation at specific points of the pathway. Additionally, we were able to observe unpredicted effects on intermediates in cells treated with ChemDiv C324-2728 (11). This assay allows for characterization of this Gram-negative pathway which is the source of the permeability barrier, LPS, presenting one of the largest challenges to antibiotic drug development. Furthermore, this assay provides a cellular context that can be used in conjunction with biochemical assays to perform target-based drug discovery and identify LPS inhibitors from phenotypic hits.

## Supporting information

S1 Fig LPS intermediate sample stability over 72 hours in neutralized SPE elution buffer. Intermediate stability was evaluated under accumulating conditions, using compound-treatment if available otherwise using genetic modification to induce accumulation.
(JPG)

S2 Fig LPS intermediate standard curves with linear fits. Standard curves are shown for each analyte with linear fits (R^2^>0.99) representing the approximate linear range of the assay for each analyte as tested with authentic standards.
(PNG)

S1 Table Strains used in this study. (DOCX)

S2 Table Plasmids used in this study [39]. (DOCX)

S3 Table Primers used in this study. Restriction sites for use in plasmid constructions are underlined.
(DOCX)

S4 Table MICs in *E*. *coli ΔtolC* and *E*. *col ΔtolC Δcdh*. MICs of select compounds were tested in the two *E*. *coli* strains according to CLSI guidelines. No difference in susceptibility was observed between the two *E*. *coli* strains.
(DOCX)

S1 File Supplementary data file: LPS metabolic perturbations. The LPS pathway inhibition heatmap (Fig 9) were generated using the analytical methods and data normalization protocols as outlined in the manuscript. All compounds were tested in dose response ranging from 8X MIC to 0.0625X MIC. The data from this table was input into Spotfire for hierarchical clustering to display similarities between accumulation and depletion profiles for these compounds. This data table is provided to support re-analysis of the dataset in the manuscript such as: algorithm training, or comparisons with compounds having other mechanisms of action.
(XLSX)
