# Supplementary figures and images for: Targeted lipopolysaccharide biosynthetic intermediate analysis with normal-phase liquid chromatography mass spectrometry
Source: PLoS One. 2019 Feb 8;14(2):e0211803. doi: 10.1371/journal.pone.0211803 (PMC6368293; doi:10.1371/journal.pone.0211803)

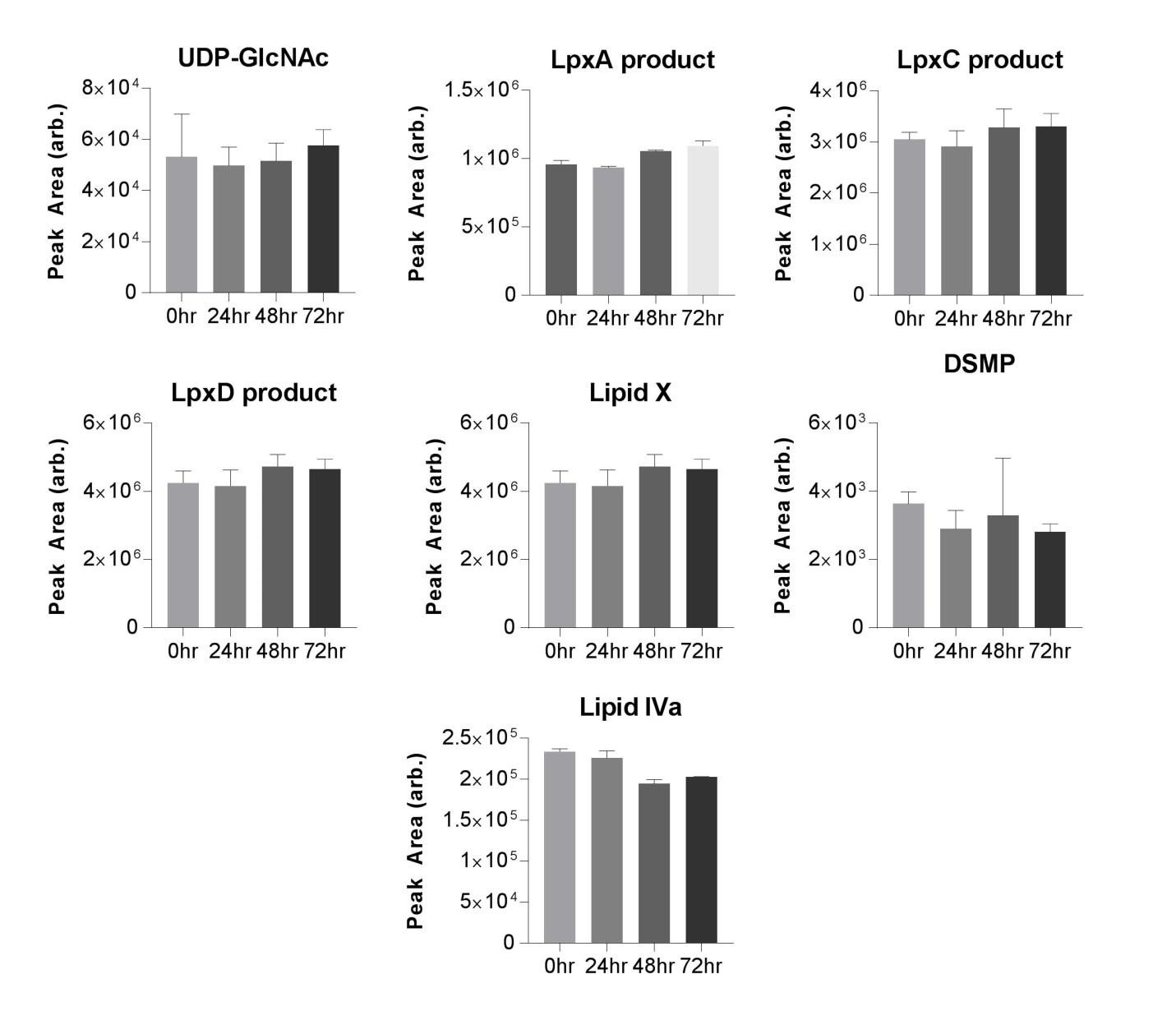

Supplement: S1 Fig — Intermediate stability was evaluated under accumulating conditions, using compound-treatment if available otherwise using genetic modification to induce accumulation. (JPG) [file pone.0211803.s001.jpg]

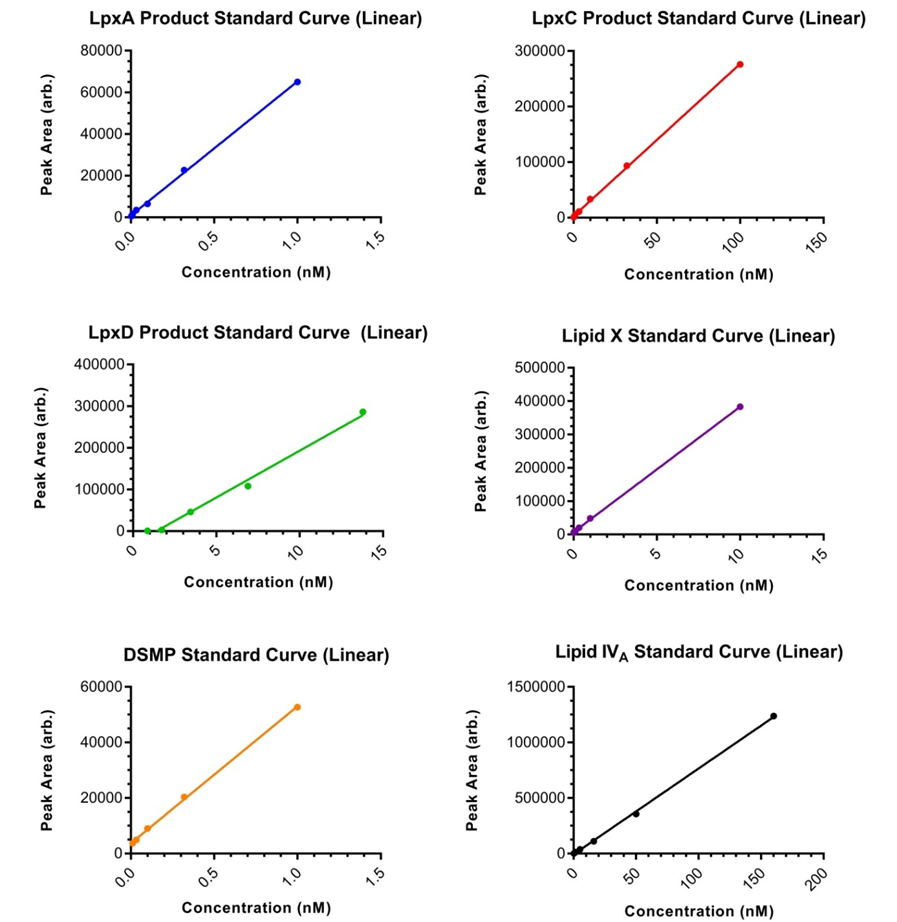

Supplement: S2 Fig — Standard curves are shown for each analyte with linear fits (R2>0.99) representing the approximate linear range of the assay for each analyte as tested with authentic standards. (PNG) [file pone.0211803.s002.png]
