# Supplementary material for: Targeted lipopolysaccharide biosynthetic intermediate analysis with normal-phase liquid chromatography mass spectrometry
Source: PLoS One. 2019 Feb 8;14(2):e0211803. doi: 10.1371/journal.pone.0211803 (PMC6368293; doi:10.1371/journal.pone.0211803)
Supplement: S1 Table — (DOCX) [file pone.0211803.s003.docx]

| Strain | Genotype | Source |
| --- | --- | --- |
| BW25113 | Δ(*araD-araB*)567 Δ*lacZ*4787(::*rrnB*-3) *rph*-1 Δ(*rhaD-rhaB*)568 *hsdR*514 | [33] |
| JW5503 | BW25113 Δ*tolC*732::Kan^R^ | [33] |
| JW3889 | BW25113 Δ*cdh*777::Kan^R^ | [33] |
| TUT0035 | BW25113 *Δcdh::frt* *ΔtolC::frt* | This study |
| TUP0005 | BW25113 *ΔlpxA*::Kan^R^ (fusion) / pTU406 (*Plac::lpxA*) | This study |
| TUP0001 | BW25113 *ΔlpxD*::frt / pTU433 (*Plac::lpxD*) | This study |
| JWM0004 | BW25113 *ΔlpxK*::Kan / pMM14 (*Plac::lpxK*) | This study |
| ClearColi K-12 | F-, λ- Δ*endA* Δ*recA* *msbA52 frr181* Δ*gutQ* Δ*kdsD* Δ*lpxL* Δ*lpxM* Δ*pagP*Δ*lpxP* Δ*eptA* | Lucigen |
