# Supplementary material for: Targeted lipopolysaccharide biosynthetic intermediate analysis with normal-phase liquid chromatography mass spectrometry
Source: PLoS One. 2019 Feb 8;14(2):e0211803. doi: 10.1371/journal.pone.0211803 (PMC6368293; doi:10.1371/journal.pone.0211803)
Supplement: S2 Table — (DOCX) [file pone.0211803.s004.docx]

| Plasmid | Genotype | Ori | Reference/source |
| --- | --- | --- | --- |
| pKD3 | *bla cat* | R6K | [27] |
| pKD13 | *bla kan* | R6K | [27] |
| pKD46 | *bla repA101(ts) araC* P*_ara_*::*γ-β-exo* | pSC101 | [27] |
| pFLP2 | Bla P*_R_*::*flp* *cI*_857_^ts^  *sacB* | pBR/colE1, ori1600 | [39] |
| pMMB206 | Cat P*_lac_*::*lacZα* | pMMB | [36] |
| pTU433 | pMMB206 P*_lac_*::*lpxD* | pMMB | This study |
| pTU457 | pMMB206 P*_lac_*::*lpxA* | pMMB | This study |
| pMM14 | pMMB206 P*_lac_*::*lpxK* | pMMB | This study |
