# Supplementary material for: Targeted lipopolysaccharide biosynthetic intermediate analysis with normal-phase liquid chromatography mass spectrometry
Source: PLoS One. 2019 Feb 8;14(2):e0211803. doi: 10.1371/journal.pone.0211803 (PMC6368293; doi:10.1371/journal.pone.0211803)
Supplement: S3 Table — Restriction sites for use in plasmid constructions are underlined. (DOCX) [file pone.0211803.s005.docx]

| Primer # | Primer description | Sequence (5’-3’) |
| --- | --- | --- |
| **TU40** | pMMB206 3' seq | GGATGTGCTGCAAGGCGATTAAG |
| **TU96** | Plac fw seq | CGCACTCCCGTTCTGGATAATG |
| **TU115** | lpxDec 5' EcoRI | GTACGAATTCAGGTTAAATAAGTAATGCCTTCAATTCGAC |
| **TU116** | lpxDec 3' HindIII | GTACAAGCTTAGTCTTGTTGATTAACCTTGCGCTC |
| **TU117** | lpxDec 5'up | CTGTGAAATCCGTTGCCAACAG |
| **TU118** | lpxDec 3'down | GCAATCGTAAGAATGAGACAGGC |
| **TU119** | lpxDec KO5' pKD13 | CATCACTGCCGACGTACTGAAACAGGTTAAATAAGTAATGATTCCGGGGATCCGTCGACC |
| **TU120** | lpxDec KO3' pKD13 | GCGAACAAAAGATGGAACGTTAGTCTTGTTGATTAACCTTTGTAGGCTGGAGCTGCTTCG |
| **TU242** | EclpxA EcoRI | ATGACCATGATTACGAATTCCGTGATTGATAAATCCGCCTTTGTG |
| **TU243** | EclpxA HindIII | GGCCAGTGCCAAGCTTTTAACGAATCAGACCGCGCGT |
| **TU263** | Ec lpxA KO F-2: | TTTGCGAAGCAACGATGATGTGTGCTCGTAGCCGGGAGGCCTGATACATGATTGAACAAGATGGATTGCA |
| **TU264** | Ec lpxA KO R-2: | GGCAATCGTTAATGGACGCTGTTCAGTCATTAACGAATCAGACCGCGCGTTTAGAAGAACTCGTCAAGAAGG |
| **TU276** | EcLpxA KO confirmation -400F | ACTGGTGGATCGCGTGCTGG |
| **TU277** | EcLpxA KO confirmation +400R | GACTGACGGACTGACGTAATG |
| **MM18** | (-)14F lpxK EcoRI | GTACGAATTCATGCAGTTTGGCCAATGATCGAA |
| **MM19** | (+)14R lpxK HindIII | GTACAAGCTTTTCATGACTCCATCAATCGAACG |
| **MM20** | lpxK pKD13 km Fw | GCGTTTACGCGCAACTTCACAAAATGCAGTTTGGCCAATGATTCCGGGGATCCGTCGACC |
| **MM21** | lpxK pKD13 km Rv | GAACGCTGCCGCGGCGTAACTAGTTGCCAGAAGCCAGCAATGTAGGCTGGAGCTGCTTCG |
| **MM22** | lpxK upstream deletion check Fw | TGAAGGCGAAATCCTGATGGATGG |
| **MM23** | lpxK ds deletion check Rv | ACGGTGGGTTAAATCATAAACGCG |
| **PT577** | Fwd primer to amplify FRT-Cm-FRT for tolC | GATCGCGCTAAATACTGCTTCACCACAAGGAATGCAAATGCATGGGAATTAGCCATGGTC |
| **PT578** | Rev primer to amplify FRT-Cm-FRT for tolC | CCTTACGTTCAGACGGGGCCGAAGCCCCGTCGTCGTCATCAGTTACGGAAAGGGTTATGGTGTAGGCTGGAGCTGCTTC |
| **PT435** | Rev primer downstream of tolC | GGAACGATGCGTGGCGTATGGATTTTGTC |
| **PT441** | Fwd primer upstream of tolC | CGATTTATCAGGGCGGAATGGTTAACTCG |
