# Supplementary material for: Targeted lipopolysaccharide biosynthetic intermediate analysis with normal-phase liquid chromatography mass spectrometry
Source: PLoS One. 2019 Feb 8;14(2):e0211803. doi: 10.1371/journal.pone.0211803 (PMC6368293; doi:10.1371/journal.pone.0211803)
Supplement: S4 Table — MICs of select compounds were tested in the two E. coli strains according to CLSI guidelines. No difference in susceptibility was observed between the two E. coli strains. (DOCX) [file pone.0211803.s006.docx]

|  |  | **MIC (µg/ml)** | |
| --- | --- | --- | --- |
| **Compound** | **Description** | *ΔtolC* | *ΔtolC*  *Δcdh* |
| CHIR-090 (9) | LpxC Inhibitor | $\leq$0.125 | $\leq$0.125 |
| ChemDiv 6359-0284 (10) | LpxD Inhibitor | 2 | 2 |
| ChemDiv C324-2728 (11) | LpxH Inhibitor | 0.125 | 0.25 |
